# Long term post PrePex male circumcision outcomes in an urban population in Uganda: a cohort study

**DOI:** 10.1186/s13104-017-2845-9

**Published:** 2017-10-30

**Authors:** M. Galukande, F. Nakaggwa, E. Busisa, D. Sekavuga Bbaale, T. Nagaddya, A. Coutinho

**Affiliations:** 1 International Hospital Kampala, P.O. Box 8177, Kampala, Uganda; 2 International Health Sciences University, Kampala, Uganda; 3 Infectious Disease Institute, Makerere University Kampala, Kampala, Uganda

**Keywords:** PrePex, Long term outcomes, Scar appearance, Sexual enjoyment

## Abstract

**Objective:**

The objective of this study was to determine the long term adverse events profile at least a year after safe male circumcision.

**Results:**

A cohort study, investigating patients who had undergone a non surgical circumcision procedure called Prepex. The study variables included scar appearance and sexual experiences. Clients were contacted for a phone interview and data were collected using a questionnaire, for some, a physical examination was done. We obtained ethical committee approval. Data from 304 out of a possible 625 men were analyzed, the rest was lost to follow up. The follow up period was 12–24 months. The mean age was 28 years. Up to 97% were satisfied with the penile scar appearance and the absence of pain. There was no keloids formation, though one developed a hypertrophic scar. Participants reported improved sexual intercourse enjoyment (post circumcision). Up to 17% resumed sexual intercourse before the 6-week long mandatory abstinence period. The average self-reported healing time was 4.7 weeks. There was a high level of scar appearance satisfaction, there was no keloids formation. There was a perceived improvement of sexual enjoyment after circumcision.

*Trial registration* ClinicalTrials. Gov Identifier: NCT02245126 (Date of registration: September 19, 2014)

## Background

PrePex like a few other non-surgical devices for voluntary medical male circumcision (VMMC) carries promise for scale up. Moreover, it has a low AE profile comparable to the conventional surgical circumcision. However, this low AE profile has been assessed for only the short and immediate term outcomes [1–3].

The prevalence of male circumcision (MC) in Uganda was 27% [4]. There are increased efforts to scale up this MC intervention which has been proven to reduce the risk of contracting HIV by 60% or more [5–7]. Many men seek to be circumcised to not only prevent HIV, but also to improve their sexual experiences [8]. While the focus is currently on scaling up voluntary medical male circumcision (VMMC), there is a need to document men’s experiences beyond the immediate post circumcision period. Since men also opt for MC not only for its protective effect against HIV acquisition but also for other reasons such as enhanced hygiene, and increased sexual pleasure [9]. Understanding men’s experiences after MC may improve our knowledge of their motivation to embrace.

International Hospital Kampala (IHK) in collaboration with the Infectious Diseases Institute (IDI) has carried out VMMC in Kampala since 2011. In 2013, a non-surgical device for circumcision (PrePex) was introduced for the first time in Uganda. The device significantly reduce the time spent on the surgical procedure, and may ultimately increase the number of men being circumcised in an effort to scale up circumcision for HIV prevention in Uganda [10].

The purpose of this paper was to describe the level of satisfaction with penile scars, healing time and post circumcision sexual enjoyment of men who had undergone PrePex circumcision after 1 year.

## Methods

### Design and setting

A male cohort study conducted in an urban, a low income country. The procedures were performed at International Hospital Kampala (IHK) a private tertiary hospital, carried out in November 2013, 1 year after the PrePex circumcisions were done.

### Sampling and data collection

Using a predesigned guide telephone interviews, were conducted with men a year after they had been circumcised to collect data. The protocol was to call at least 3 times per week for 2 consecutive weeks before delaying the individual unreachable and therefore drops out of the participants list. Only data from men who responded to the telephone calls and completed the questionnaire were included in the study.

### Study variables

The study variables included self reported sexual intercourse enjoyment; defined in comparison to the pre-circumcision status the experiences as better or worse/same (dichotomous key). Other question items included pain at intercourse, premature ejaculation and difficult vaginal penetration during intercourse. Subjective scar appearance defined in terms of scar thickness, contour, texture and color. We asked questions such as, ‘Is the scar in your opinion neat?’, ‘Is the scar smooth?’, ‘Is the scar well blended?’. Considering these parameters the respondent scored it as satisfactory or not satisfactory (dichotomous key). The scar assessment parameters used were drawn from previous guides [11, 12].

Sexually active was defined as engaging in sexual activities involving penetrative sex.

### Data analyses

Using a precoded and pretested questionnaire during the phone interviews, we gathered the respondents’ demographic characteristics, scar appearance and sexual experiences before and after circumcision. Using logistic regression, odds ratio (OR) were calculated as a measure of association between time of resumption of sex and the healing time. Marital status and religion were included. Significant p value was < 0.05. These data were entered and analyzed using SPSS V. 20.

### Inclusion and exclusion criteria

We included all clients that had undergone a voluntary PrePex circumcision during the study period. We excluded those (five) who had PrePex initially and followed up by a surgical circumcision as an intervention for an adverse event.

## Results

Of the 634 men who enrolled for the non surgical PrePex method of circumcision, 304 were enrolled in this evaluation after attempts to contact all of them were made. Failed attempts were due to: unanswered calls, wrong numbers or phones persistently (2 weeks) switched off. Participants in the age group 26–35 constituted the highest percentage of respondents at 56.6% (172) followed by those below 25 years that constituted 33.6%. The mean age was 28.4 years; majority of the respondents were single (58.2%) while 41.1% were married at the time of the interview. Of those who were married, 16 (5.3%) had been single at the time of circumcision (see Table 1).

**Table 1:** Participants’ socio-demographic characteristics and pre circumcision sexual experiences

	Frequency	Percentage
Characteristic		
18–19	6	2.0
20–24	73	24.0
25–29	122	40.1
30–34	67	22.0
35 +	36	11.8
Religion		
Protestant	119	39.1
Catholic	133	43.8
Muslim	3	1.0
Pentecostal	41	13.5
Other	8	2.6
Education level		
None	2	0.7
Primary	26	8.6
Secondary	124	40.8
Tertiary	152	50.0
Marital status		
Single	177	58.2
Married/cohabiting	125	41.1
Separated	2	0.7
Place of residence		
Makindye	153	50.3
Kawempe	25	8.2
Rubaga	15	4.9
Central division	13	4.3
Nakawa	82	27.0
Other	16	5.3
Total	304	100
Pre MC sexual experiences		
Premature ejaculation	75	29
Pain on penetration	81	31
Friction on penetration	65	25
Delayed ejaculation	17	6.5
Penile sensitivity	4	1.5
Pain on erection	6	2
Others	13	5
Total	261	100

### Healing

The average self-reported healing time was 4.7 weeks; 256 (84.2%) of the respondents healed within 6 weeks while 47 (15.5%) healed between 6 and 32 weeks. In total, 99.7% reported complete healing a year after circumcision. Only 1 person (0.3%) said they had not healed completely because he still felt pain during sexual intercourse he never felt pain before circumcision.

Neither marital status before circumcision nor marital status after circumcision were not statistically significant for determining whether or not a respondent healed within 6 weeks (see Table 2).

**Table 2:** Healing time versus demographic characteristics

	Total (N = 304)	Healing in 6 weeks		*χ* ^2^, p value
		No n (%)	Yes n (%)	
Age category				
18–19	6	0	6 (100)	0.544
20–24	73	8 (11)	65 (89)	
25–29	122	22 (18)	100 (82)	
30–34	67	13 (19.4)	54 (80.6)	
35 +	36	05 (13.9)	31 (86.1)	
	**Total (n** **=** **304)**	**No (n** **=** **48)**	**Yes (n** **=** **256)**	
Age category				
18–24	79	8 (10.1)	71 (89.9)	0.544
25–29	122	22 (18)	100 (82)	
30–34	67	13 (19.4)	54 (80.6)	
35 +	36	05 (13.9)	31 (86.1)	
Marital status				
Unmarried	179	26 (14.5)	153 (85.5)	0.469
Married	125	22 (17.6)	103 (82.4)	
Education level				
Below secondary	28	05 (17.9)	23 (82.1)	0.946
Secondary	124	19 (15.3)	105 (84.7)	
Tertiary	152	24 (15.8)	128 (84.2)	
Religion				
Protestant	119	20 (16.8)	99 (83.2)	0.033
Catholic	133	20 (15.0)	113 (85.0)	
Pentecostal	41	03 (7.3)	38 (92.7)	
Other	11	05 (45.5)	06 (54.5)	
Residence				
Makindye	154	26 (16.9)	128 (83.1)	0.882
Kawempe	25	03 (12.0)	22 (88.0)	
Rubaga	15	02 (13.13)	13 (86.7)	
Central	13	03 (23.1)	10 (76.9)	
Nakawa	82	11 (13.4)	71 (86.6)	
Other	15	02 (20.0)	12 (80.0)	
Sex before 6 weeks	261^a^	215 (82.4)	46 (17.6)	0.021

^a^The sexually active participants

### Scar appearance satisfaction

Overall, 273 (90%) were very satisfied or extremely satisfied with scar appearance. In all, 297 (97.7%) respondents liked the scar appearance (color, texture and thickness) of their scar while 2.3% (7) said that they did not like the appearance of their scar. 116 (38%) described the scar as ‘neat’, 101 (33%) described it as even and smooth in contour and texture and 87 (29%) described it as well blended with the rest of the skin (color).

Those who disliked the appearance of their scars said that it was because the scars were uneven (3), the scar had sores (1), the scar was thick (1), and the scar being hard on touch (texture) (1) or too much skin had been removed (1).

### Sexual experiences

Before circumcision, 91.4% (278) respondents had ever been sexually active, 85.9% of the respondents reported being sexually active at the time of the interviews. Of all the respondents who were sexually active, 110 (36.2%) had had their first sexual encounter after circumcision with their wives, 138 (45.4%) with regular partners and 13 (4.3%) with new partners. Of those who had sexual encounters with new partners, 5 said that these had been female sex workers. The average number of weeks that newly circumcised men waited before having their first sexual intercourse was 11.2 weeks. Of the 261 (85.9%) respondents who were sexually active, 53 (17.6%) had not waited for the required 6 weeks before their first intercourse post circumcision while 250 (82.4%) had waited at least until the 6 weeks elapsed.

In total, 256 (84%) respondents reported complete healing within the expected 6 weeks while 48 (16%) did not heal within the 6 weeks (see Table 3). There was a statistically significant relationship between sexual intercourse before the stipulated 6 weeks and whether healing occurred within that time (p = 0.02). Respondents who had perceived themselves as healed had a high likelihood of resuming sexual intercourse before the mandatory 6 week period (unadjusted OR = 4.8: 95% CI 1.1, 20.4).

**Table 3:** Associations between men’s characteristics and being healed before 6 weeks

Characteristic	Unadjusted OR (95% CI)	p value	Adjusted OR (95% CI)	p value
Sex before 6 weeks				
No	1		1	
Yes	4.8 (1.1–20.4)	0.035	6.4 (1.4–29.0)	0.016
Marital status				
Unmarried	1		1	
Married	0.9 (0.4–1.5)	0.470	0.7 (0.4–1.3)	0.220
Religion				
Protestant	1		1	
Catholic	1.1 (0.6–2.2)	0.701	1.2 (0.6–2.3)	0.681
Pentecostal	2.6 (0.7–9.1)	0.147	2.9 (0.8–10.4)	0.103
Other	0.2 (0.1–0.9)	0.030	0.2 (0.0–0.7)	0.014

In all, 233 (76.6%) of the respondents reported improved sex life after circumcision while 26 reported no improvement in their sex life; 3 people reported deterioration in their sex life post circumcision.

Before they were circumcised, 261 participants reported pain on penetration, premature ejaculation and friction on penetration (see Table 1).

Among the changes in sexual experiences smooth penetration and prolonged ejaculation were mentioned. Smoother penetration 129 (42.2%), prolonged ejaculation 95 (31.5%), heightened glans sensitivity 56 (18.4%), lowered glans sensitivity 19 (6.2%) and others 5 (1.5%).

Forty-six (15%) respondents reported that their sex life had improved because their sexual partners found their sex experience after circumcision more fulfilling.

*“My wife appreciates that I take longer to ejaculate” Respondent. “I sustain an erection for longer so my wife enjoys sex” Respondent*

## Discussion

We set out to investigate the long-term cosmetic and sexual experiences post PrePex circumcision. We found a high level of satisfaction with the outcomes, similar to a ShangRing device study by Feldblum et al. in Kenya [13]. Other non-device (surgical) post circumcision surveys in Uganda and Kenya suggested high levels of satisfaction too [14, 15]. The majority were satisfied with the scar look. The scar formed by a device such as PrePex is devoid of stitch marks, stitch marks tend to give an unsatisfactory look, and surgical circumcision is prone to uneven circular lines. One client complained of a hard scar, when reviewed he had the features of a hypertrophic scar. Another complained of sores superimposed onto the scar, there are some possibilities, including an STI, or simply a broken down scar, the actual reason was not verified.

Reporting too much skin removal normally leads to a buried or shortened penis, and painful erections, this was the first one in this cohort over the 12–18 month period, removing much is an error in marking the point/level at which the rubber ring is loaded or sits.

Even though the main purpose for voluntary male medical circumcision is partial HIV prevention, it appears that there other substantial benefits that may be a motivator for the clients. The reported improved penetration is likely not only to be a function of circumcision, but also adequate vaginal lubrication, it is not clear whether circumcision per se would indirectly lead to better anticipation on part the of the spouse and therefore better lubrication and eased penetration.

Delayed ejaculation may in part be explained by reducing glans sensitivity, but perhaps there could be psychological effects too. As indicated by some, being circumcised is a desirable state. Yet for others the heightened state of glans sensitivity had been desirable.

A significant proportion did not wait for the required 6 weeks, perhaps on the assumption that they had completely healed from the lack of pain and no open wound. In Kenya study in 2013, the risk factors for sex before healing were being married or having 2 or more sex partners [16]. In this study marital status was a not significant risk factor. The number of sexual partners was not evaluated. Sexual intercourse resumption before complete healing has programmatic implications because it exposes the individuals to risk of STIs even though only a small number engaged in clearly high-risk sex with sex workers, we weren’t certain if they had used condoms. The message should be 6 weeks of abstinence irrespective of whether one deems themselves healed [17].

The strength of this paper is it provides data on long term outcomes of safe male circumcision device that has not been studied much.

## Limitations

As there was no comparison group, this limited the inferences regarding PrePex, however, we can conclude that PrePex per had a high level of satisfaction. While the PrePex cohort provided insight into men’s post circumcision experiences, there is a need to compare these experiences with a cohort of men who were surgically circumcised. Other benefits of circumcision should be incorporated into health education and promotion activities. Women’s involvement is important in scaling up circumcision as several men felt that their sexual experiences after circumcision made their partners more satisfied sexually.

Loss to follow-up was a major challenge for young urban or peri-urban clients as they are highly mobile and difficult to contact. The reasons for failure to reach everyone were related to inability to reach them by phone which reasons are possibly not related to the outcomes we looking for perhaps the study group is representative of the entire cohort.

## Conclusions

There was a high level of scar appearance satisfaction; there was no keloids formation, however, there was a hypertrophic scar formation. There was a perceived improvement of sexual enjoyment after circumcision. The average self-reported healing time was 4.7 weeks. This was associated with a significant proportion (17%) resuming sexual intercourse before the required 6 weeks.

## Abbreviations

AE: adverse event
HIV: human immunodeficiency virus
IDI: Infectious Diseases Institute
IHK: International Hospital Kampala
STI: sexually transmitted infections
VMMC: voluntary male medical circumcision
